# Evaluation of Peripheral Retinal Changes on Ultra-widefield Fundus Autofluorescence Images of Patients with Age-related Macular Degeneration

**DOI:** 10.4274/tjo.galenos.2019.00359

**Published:** 2020-03-05

**Authors:** Kübra Küçükiba, Nazmiye Erol, Muzaffer Bilgin

**Affiliations:** 1 Eskişehir Osmangazi University Hospital, Clinic of Ophthalmology, Eskişehir, Turkey

**Keywords:** Age-related macular degeneration, autofluorescence, peripheral abnormalities, ultrawide-field imaging

## Abstract

**Objectives:**

Age-related macular degeneration (AMD) is the most common cause of central vision loss in individuals aged 65 years and older in developed countries. Earlier imaging systems did not enable visualization of the peripheral retina in diseases affecting the macula. With the introduction of new-generation devices, the peripheral retina is easily visualized. In our study, we aimed to evaluate the incidence of peripheral retinal changes in the color and autofluorescence fundus images of patients with AMD.

**Materials and Methods:**

In the study group, 550 eyes of 277 patients who were diagnosed with AMD and 90 eyes of 45 healthy patients in the control group were evaluated. An ultra-wide-angle imaging device was used to record standard 200° color and autofluorescence fovea-centered fundus images followed by superior and inferior fundus images obtained using the device’s fixation light. The fundus images were examined in 3 sections: zone 1, zone 2, and zone 3.

**Results:**

Evaluation of color fundus images revealed peripheral retinal changes in 67.8% of the 550 AMD eyes and 47.8% of the healthy eyes. Drusen was the most common peripheral retinal change. Evaluation of autofluorescence images revealed peripheral autofluorescence changes in 39.6% of the AMD eyes and 28.9% of the healthy eyes. Hypoautofluorescence was the most common autofluorescence change.

**Conclusion:**

Peripheral retinal changes were more common in AMD patients than the control group, indicating that AMD is not only a macular disease, but can affect the entire retina. Future prospective studies will elucidate the relationship between these peripheral retinal changes and patients’ genetic features and their importance in prognosis, diagnosis, and treatment.

## Introduction

Age-related macular degeneration (AMD) is the most common cause of central vision loss among individuals aged 65 years and older in developed countries.^1^ While hyperpigmentation of the retinal pigment epithelium (RPE) and lipofuscin deposition in the macula are the earliest signs of the disease, there are studies demonstrating that findings such as drusen and pigmentary changes in the retina are present not only in the macula but also in the peripheral retina.^2,3,4,5^

Thanks to advances in imaging methods, high-resolution images of the central and peripheral retina have been acquired since the 2000s using ultra-wide-angle imaging systems that can capture a 200° section of retina. With peripheral retinal imaging, color and autofluorescence images revealed that peripheral retinal changes occurred in extramacular areas in AMD patients. Color and autofluorescence images demonstrated that more peripheral retinal changes take place in patients with AMD compared to healthy individuals.^4,5^

The aim of the present study was to determine and compare the prevalence of peripheral retinal changes in color and autofluorescence images in healthy individuals and AMD patients.

## Materials and Methods

This prospective analysis included 550 eyes of 277 patients who presented to the Ophthalmology Department of the Eskişehir Osmangazi University Medical Faculty Hospital between June 2016 and July 2017 and were diagnosed with AMD, as well as 90 eyes of 45 patients with healthy retinas. Ethics committee approval was obtained before the study (number 980558721/137, dated May 30, 2016).

The 277 patients in the AMD group were selected from patients who were over 55 years of age, had no more than 6 diopters of refractive error, had not undergone retinal surgery or laser photocoagulation, and had no history of retinal disease other than AMD. AMD was diagnosed by evaluating the patients’ fundus examinations and optical coherence tomography (OCT) images.

The 45 healthy individuals in the control group were over 55 years of age, had no more than 6 diopters of refractive error, had not undergone retinal surgery or laser photocoagulation, and had no history of any retinal disease.

After being informed about the study, all patients signed written consent forms. The age and gender of the patients in the AMD and control groups were recorded. All patients’ pupils were dilated by instilling 2.5% phenylephrine and 1% tropicamide drops into both eyes. OCT images were acquired after dilation. An ultra-wide-angle Optos 200 Tx device was used to take 200° standard fovea-centered color images of both eyes, followed by superior and inferior fundus images obtained using the device’s fixation light. Fundus autofluorescence images were acquired and recorded following the same protocol.

### Image Evaluation

**Figure 1:**
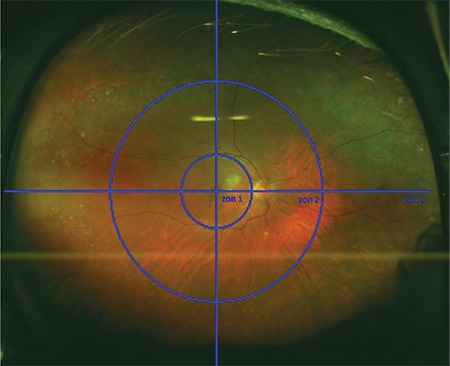
Zones 1, 2, and 3 on the fovea-centered image

The obtained images were viewed using the Optos V2 Vantage Pro Review software. Fovea-centered, superior, and inferior color and autofluorescence images were acquired, for a total of 6 different images for each eye. All images were evaluated together with an experienced retina specialist. Evaluation began with fovea-centered images. Color fundus images were evaluated in 3 sections (zone 1, zone 2, and zone 3), as described in previous studies (Figure 1).^4,5,6^

Zone 1 was defined as the area within a 5.4 mm diameter centered on the fovea and including the nasal edge of the optic disc and the temporal macula. It corresponds to approximately 3 optic disc diameters (DD). For AMD patients, zone 1 images were evaluated together with OCT images AMD grading was done according to AREDS Research Group criteria.^7^ AMD was graded as early in the presence of drusen smaller than 125 µm, intermediate if drusen were larger than 125 µm, and late in the presence of findings characteristic of geographic atrophy or neovascular AMD. Patients with late AMD were further divided into neovascular and geographical atrophy subgroups.

**Figure 2:**
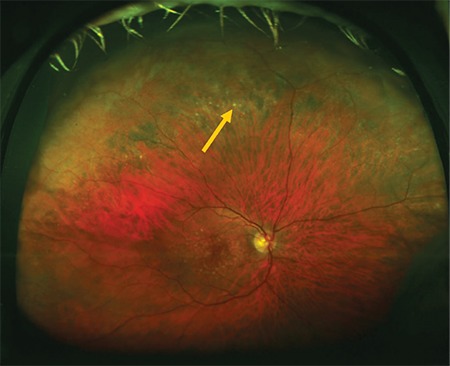
Color fundus image acquired with upward gaze fixation showing peripheral drusen (arrow)

**Figure 3:**
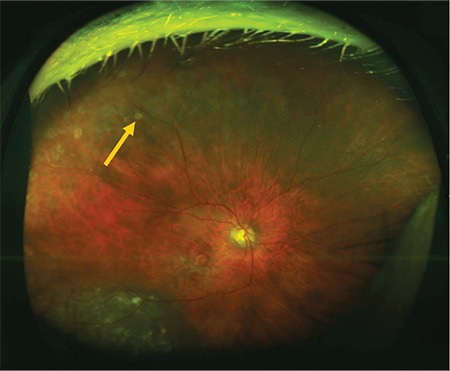
Color fundus image acquired with upward gaze fixation showing peripheral RPE hypopigmentation (arrow)

**Figure 4:**
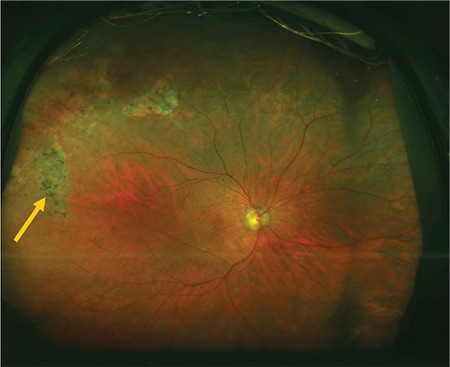
Fovea-centered color fundus image showing RPE hyperpigmentation (arrow)

**Figure 5:**
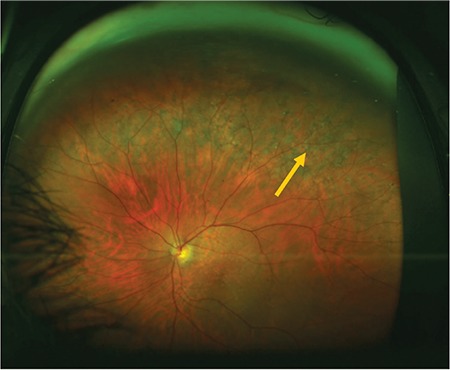
Color fundus image acquired with upward gaze fixation showing reticular changes (arrow)

**Figure 6:**
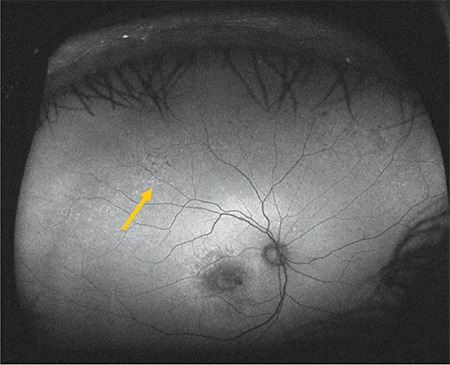
Fundus autofluorescence image acquired during upward gaze fixation showing hyperautofluorescence (arrow)

**Figure 7:**
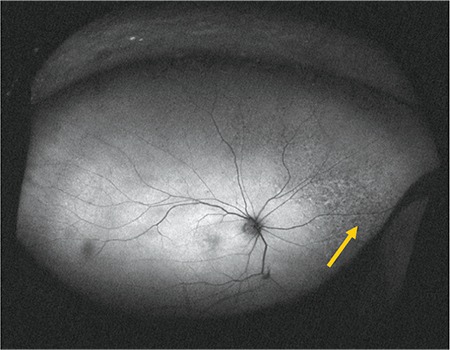
Fundus autofluorescence image acquired during upward gaze fixation showing hypoautofluorescence (arrow)

**Figure 8:**
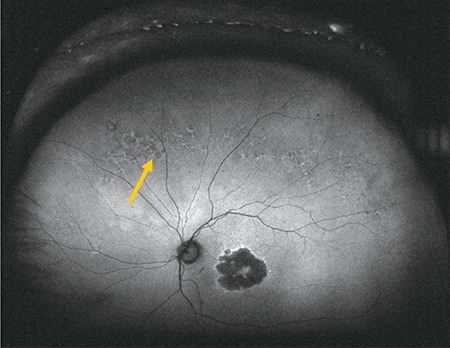
Fundus autofluorescence image acquired with upward gaze fixation showing a halo (lesion with hyperautofluorescent ring surrounding a hypoautofluorescent center) (arrow)

Zone 2 was defined as the area within a 16.2 mm circle, equivalent to 9 DD. Its inner boundary starts from zone 1 and its outer boundary coincides with the vortex veins. Changes observed in zone 2 color fundus images were recorded as drusen (Figure 2), RPE hypopigmentation (Figure 3), RPE hyperpigmentation (Figure 4), and reticular changes (Figure 5). Changes observed in zone 2 FAF images were recorded as hyperautofluorescence (Figure 6), hypoautofluorescence (Figure 7), and a halo (a lesion with a hypoautofluorescent center surrounded by hyperautofluorescence) (Figure 8).

**Figure 9:**
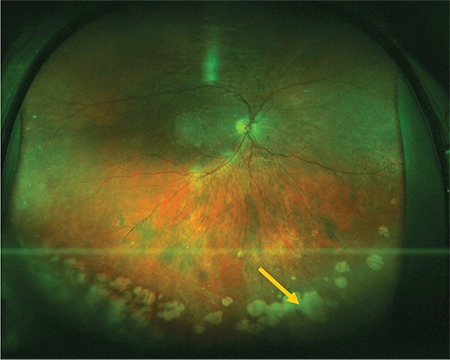
Color fundus image acquired with downward gaze fixation showing cobblestone degeneration (arrow)

**Figure 10:**
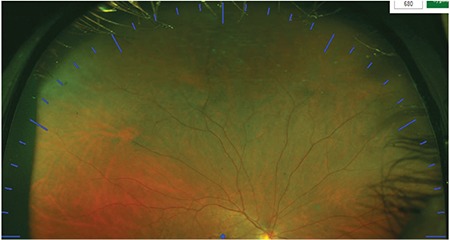
Clock-hour division of the retina in images acquired with upward gaze fixation

**Figure 11:**
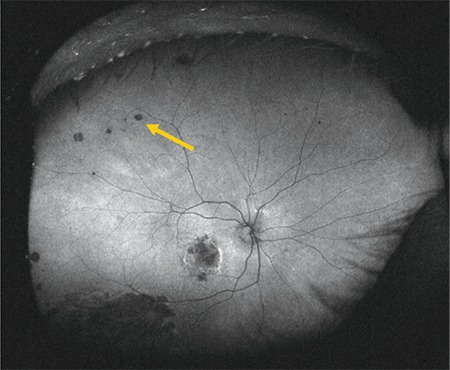
Fundus autofluorescence image acquired with downward gaze fixation showing a nummular lesion (arrow)

**Figure 12:**
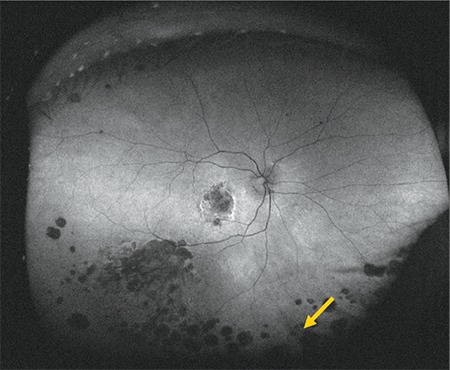
Fovea-centered fundus autofluorescence image showing cobblestone autofluorescence (arrow)

Zone 3 was defined as the peripheral retina beyond zone 2 and was divided into 180° sections. Superior images obtained with upward gaze fixation were used to evaluate the upper half and inferior images obtained with downward gaze fixation were used to evaluate the lower half. Drusen, RPE hypopigmentation, RPE hyperpigmentation, reticular changes, cobblestone degeneration (Figure 9), and other changes observed in zone 3 color images were recorded. The area covered by the lesion was expressed in degrees by dividing the retina into clock hours (Figure 10). In eyes with multiple lesions, the largest was recorded as the primary lesion. Changes in zone 3 FAF images were recorded as hyperautofluorescence, hypoautofluorescence, halo, nummular (small, round hypoautofluorescent lesion with smooth border) (Figure 11), and cobblestone (multiple medium to large round hyperautofluorescent lesions with smooth borders) (Figure 12). Similar to the protocol used for color images, the area covered by the lesions was recorded in degree according to clock hour and the largest of multiple lesions was recorded as primary.

After zones 1 and 2 were evaluated based on fovea-centered images as shown in Figure 1, zone 3 was evaluated based on upward and downward gaze images.

An image was considered acceptable for analysis if the entirety of zone 1 and zone 2 and more than 50% of zone 3 were visible. Other images were excluded from the study.

### Statistical Analysis

All data were entered into Microsoft Office 2013 Excel software. Continuous data were expressed as mean ± standard deviation. Categorical data were expressed as percentage (%). The Shapiro-Wilk test was used to determine whether the data were normally distributed. When comparing groups with normal distribution, independent samples t-test was used in comparisons of 2 groups and one-way analysis of variance (ANOVA) was used for 3 or more groups. Pearson’s chi-square and Fisher’s exact tests were used to analyze the resulting contingency tables. IBM SPSS Statistics 21.0 software was used to perform the analyses. A p value <0.05 was accepted as the criterion for statistical significance.

## Results

In this study we evaluated 550 eyes of 277 patients diagnosed with AMD and 90 eyes of 45 healthy individuals in the control group. Four eyes in the AMD group that did not meet the evaluation criteria were excluded from the study. The age and sex distributions of patients in the AMD patients and control groups are shown in Table 1.

In the AMD group, 92 eyes (16.7%) were graded as early stage, 99 eyes (18%) as intermediate stage, and 359 eyes (65.3%) as late stage. Of those in the late AMD group, 95 eyes were evaluated as having geographic atrophy (17.3%) and 264 eyes as having neovascular AMD (48%).

### Evaluation of Color Fundus Images

When color fundus images were evaluated, peripheral retinal changes in zone 2 and/or zone 3 were observed in 67.8% of the 550 eyes in the AMD group and in 47.8% of the 90 eyes in the control group. There were significantly more peripheral retinal changes in the AMD group compared to the control group (p<0.001).

The rates of the peripheral retinal changes detected when the color images of all patients in the AMD and control groups were evaluated are shown in Table 2.

When all peripheral zones were evaluated, rates of peripheral retinal change were significantly higher in the AMD group compared to the control group.

The distribution of peripheral retinal changes observed in the color fundus images of subjects in the AMD and control groups according to lesion type is shown in Table 3.

Drusen were the most common peripheral retinal change. Rates of drusen detection in zone 2, superior zone 3, and inferior zone 3 images were 29.6%, 54.2%, and 40.9%, respectively in the AMD group and 18.9%, 30%, and 21.1%, respectively in the control group (p=0.042, p<0.001, p<0.001). Compared to the control group, the AMD group had significantly more drusen in all areas.

When pigmentary changes were evaluated, the prevalence of RPE hypopigmentation in zone 2, superior zone 3, and inferior zone 3 was 3.1%, 6%, and 9.3% in the AMD group and 0%, 3.3%, and 4.4% in the control group, respectively (p=0.15, p=0.441, p=0.189). Rates of RPE hyperpigmentation in the AMD and control groups were 3.1% and 0% for zone 2, 2.7% and 2.2% for superior zone 3, and 5.5% and 4.4% for inferior zone 3, respectively (p=0.150, p=1.0, p=1.0). The prevalence of reticular changes in the AMD and control groups was 0.4% and 0% for zone 2, 4% and 0% for superior zone 3, and 5.3% and 3.3% for inferior zone 3, respectively (p=1.0, p=0.058, p=0.604). There was no statistically significant difference between the groups in terms of pigmentary changes.

The prevalence of cobblestone degeneration, which was evaluated under other peripheral changes, in superior and inferior zone 3 was 1.5% and 6.9% in the AMD group and 1.1% and 3.3% in the control group, respectively (p=1.0, p=0.293). There was no statistically significant difference between the groups.

When evaluating the color fundus images of subjects in the AMD and control groups, the largest of multiple lesions was identified as the primary lesion and others as secondary lesions. Analysis of superior zone 3 images of the 550 eyes in the AMD group revealed primary peripheral retinal changes in 338 eyes (61.4%). Drusen were the most common primary lesion (82.5%), followed by RPE hypopigmentation (6.8%), reticular changes (5.6%), RPE hyperpigmentation (3.6%), and cobblestone degeneration (1.5%). Peripheral retinal changes in inferior zone 3 were detected in 294 eyes (53.4%). Similar to superior zone 3, drusen were the most common lesion (64.6%). RPE hypopigmentation and cobblestone degeneration were observed at equal rates (11.2%), followed by reticular changes (7.1%) and RPE hyperpigmentation (5.8%). The most common primary peripheral retinal change observed in superior and inferior zone 3 of patients in the control group was also drusen.

The most important and nonmodifiable risk factor associated with AMD is age. As mentioned in previous studies, we compared patient subgroups with no statistical age difference in order to prevent a miscalculation that may result from differences in age.^6^ The analysis included AMD patients between 65 and 79 years of age and control subjects between 60 and 71 years of age. Peripheral retinal changes were detected in 67% of 306 eyes in the AMD subgroup and in 34% of 50 eyes in the control subgroup. Based on this age-matched comparison, there were more significantly more peripheral retinal changes in the AMD group compared to the control group (p<0.001).

We also divided the AMD patients into subgroups based on disease stage and made comparisons between them and with the control group. Compared to the control group, patients with early, intermediate, and late AMD all showed significantly higher rates of peripheral retinal changes (p=0.038, p=0.001, p=0.001).

In the AMD group, the area occupied by lesions in the superior and inferior quadrants was measured in degrees. The largest lesion type in the superior quadrants was reticular changes, while the largest lesion type in the inferior quadrants was cobblestone degeneration.

### Evaluation of Fundus Autofluorescence (FAF) Images

When FAF images were evaluated, peripheral autofluorescence was observed in zone 2 and/or zone 3 in 39.6% of the 550 eyes in the AMD group and 28.9% of the 90 eyes in the control group. This difference between the groups was not statistically significant (p=0.052).

The prevalence of peripheral autofluorescence detected in the FAF images of all patients in the AMD and control groups is shown in Table 4.

The distribution of the peripheral retinal changes in the FAF images of patients in the AMD and control groups are shown in Table 5.

There was no statistically significant difference between the groups in the prevalence of hyperautofluorescence in zone 2 (p=0.002) or in superior and inferior zone 3 (p=0.394, p=1.00).

The prevalence of hypoautofluorescence in zone 2, superior zone 3, and inferior zone 3 were 8.4%, 14.4%, and 12.2% in the AMD group and 0%, 11.1%, and 10% in the control group, respectively (p=0.009, p=0.508, p=0.676). There were significantly more hypoautofluorescence lesions in zone 2 in the AMD group compared to the control group (p=0.004). Nummular autofluorescence was observed in superior and inferior zone 3 in 3.5% and 6.5% of eyes in the AMD group and 0% and 1.1% of eyes in the control group, respectively (p=0.092, p=0.071). The prevalence of cobblestone autofluorescence in superior and inferior zone 3 images was 1.5% and 6.9% in the AMD group and 1.1% and 3.3% in the control group, respectively (p=1.0, p=0.293). There were no statistically significant differences between the groups in terms of nummular or cobblestone autofluorescence rates.

Evaluation of superior zone 3 in the 550 AMD eyes revealed primary peripheral autofluorescence change in 163 eyes (29.6%). Hypoautofluorescence was the most common change (44.8%), followed by hyperautofluorescence (32.5%), halo (12.9%), and nummular lesions (7.4%). Cobblestone autofluorescence was the least common peripheral autofluorescence change (2.5%). Peripheral autofluorescence changes in inferior zone 3 were observed in a total of 178 eyes (32.3%). In inferior zone 3, hypoautofluorescence was the most common change (28.7%), followed by hyperautofluorescence (25.3%) and cobblestone (20.2%), nummular (14%), and halo hypoautofluorescence (11.8%).

As with color images, statistically age-matched subgroups were compared when evaluating FAF images. Peripheral autofluorescence changes were detected in 36.9% of 306 eyes in the AMD subgroup and 14% of 50 eyes in the control subgroup. Peripheral autofluorescence changes were significantly more common in the age-matched AMD subgroup compared to the control group (p=0.003).

Again, we also made comparisons between the control group and AMD subgroups based on disease stage. There were no significant differences in the prevalence of peripheral autofluorescence changes in patients with early, intermediate, and late AMD compared to the control group (p=0.394, p=0.097, p=0.054). We further subclassified patients with late AMD into those with geographical atrophy and neovascular for comparison. There was a significant difference between these groups in terms of the prevalence of peripheral autofluorescence change (p=0.042), with more peripheral autofluorescence change in the geographical atrophy subgroup (49.5%) than in the neovascular subgroup (37.5%).

In the AMD eyes, the area occupied by lesions in the superior and inferior quadrants was measured in degrees. The largest lesion type in both hemispheres was hyperautofluorescence.

Color and FAF images of patients in the AMD group were examined in terms of the superior and inferior zone 3 regions. The comparison of rates of all retinal changes detected in superior and inferior zone 3 is shown in Table 6.

Peripheral retinal changes detected in color fundus images were significantly more common in the superior region compared to the inferior region (p<0.001). However, peripheral autofluorescence changes were significantly more common in the inferior region (p<0.001).

## Discussion

Due to advances in imaging methods, we can now obtain peripheral retinal images using ultra-wide-angle imaging systems. In patients with AMD, the peripheral retina lying beyond the macula can be evaluated easily with color and autofluorescence images. Imaging of the peripheral retina has shown that peripheral retinal changes also occur in extramacular areas of the retina in AMD.

In this study comparing the color fundus and FAF images of AMD patients with those of healthy subjects, we evaluated similar studies in the literature and presented a detailed explanation of lesion names, locations, and evaluation methods in order to provide a summary of the interpretation and classification of these changes and to facilitate the standardization of future studies conducted in this field.

A review of the literature shows that the first study conducted with an ultra-wide-angle imaging system was published by Reznicek et al.^8^ in 2012. The study investigated peripheral autofluorescence intensity and abnormalities in patients with AMD. A significant difference was observed between the AMD and control group in terms of peripheral autofluorescence changes. The results of that study demonstrated that lipofuscin deposition in the RPE in AMD patients occurred not only in the macula, but also in the peripheral retina. However, only intensity was measured from the peripheral FAF images; the peripheral lesions and their features were not described.^8^

Since more standard images were obtained in published studies that utilized the software developed by Optos to eliminate torsion in images obtained in the upward and downward gaze fixation positions of the device, these studies seem to have less subjectivity.^4,9^ In the present study, we evaluated images taken without using such software separately. In future studies, it may be beneficial to use software that ensures standardization in order to prevent analytical errors that may result from subjective evaluation.

Because there is no indicator that would ensure standard image acquisition, the images appear larger than they actually are if the patient gets too close to the device and smaller than they actually are if the patient is too far from the device. For this reason, the size of the patient’s optic disc was used as a reference when evaluating each image in an effort to prevent the errors that can arise in millimetric measurements, which led to the concept of zones. Studies conducted to date present different views regarding how the midperipheral area (i.e., the retinal region referred to as zone 2) should be calculated.^4,5,7,8,9,10,11,12^ When planning the present study, we accepted an area of 3 DD as zone 1 and an area of 9 DD as zone 2 (considered the midperiphery).^5,6^ Confusion regarding the definition of the midperipheral region in the literature is preventing its standardization in current studies and necessitates the establishment of standard measurements which can be incorporated into emerging technologies.

There are also differences of opinion regarding the identification of autofluorescence changes in the periphery and midperiphery. Every change observed in the present study was named based on commonly used definitions in previous studies and is described together with an image. In one study, the rate of consistency between two independent researchers evaluating FAF images was reported as 78.4%.^13^ In the same study, when the researchers were asked to differentiate between hypoautofluorescence and hyperautofluorescence, the agreement rate fell to 69%. Ultimately, the interpretation of FAF images is subjective. Although increasing the number of researchers improves reliability, it does not ensure standardization.

In our evaluation of AMD patients, peripheral retinal changes were observed in 67.8% of their color fundus images and 39.6% of their FAF images. The prevalence of peripheral autofluorescence changes was 29.6% in superior images and 32.4% in inferior images. More peripheral retinal changes were detected in the color fundus and FAF images of AMD patients compared to healthy subjects.

Drusen was the most common peripheral retinal change observed in color images of zone 2, superior zone 3, and inferior zone 3 in both the AMD and control groups, while hypoautofluorescence was the most common peripheral autofluorescence change observed in FAF images. Studies in the literature present varying results regarding autofluorescence changes detected in the peripheral retina. In the OPERA study, consistent with our findings, the most common type of lesion observed in zone 2, superior zone 3, and inferior zone 3 color images from the patient and control groups was drusen, with the most common peripheral autofluorescence change observed in FAF images was hypoautofluorescence.^4^ In a study by Suetsugu et al.^12^, the most common autofluorescence change observed in the periphery (45.5%) was mottled autofluorescence (areas of irregular hypoautofluorescence). In another study, drusen was reported as the most common lesion in color images and granular (hyperautofluorescent areas) autofluorescence was the most common autofluorescence change in FAF images from the neovascular, geographic atrophy, and control groups.^5^

Drusen stands out as the most common type of peripheral lesion in studies. In a recent study, the CFHY402H genotype was linked to peripheral drusen and the CFHrs1410996 genotype to peripheral retinal pigmentary changes in patients with AMD.^14^

When the studies in the literature are compared, each research group seems to have described autofluorescence lesions in a distinctive way. In one study, lesions are described as being granular, nummular, and mottled, while in another study they are described as focal pinpoint, granular, patchy, and reticular.^5,10^ In the OPERA study, the detected lesions were described as hypoautofluorescence, hyperautofluorescence, and reticular autofluorescence.^4^ Based on our review of all relevant studies, we classified autofluorescence lesions in the present study under 5 categories: hypoautofluorescence, hyperautofluorescence, halo, nummular, and cobblestone autofluorescence.

A limitation of our study which is common to all studies is the age difference between the study and control groups. This difference may stem from the fact that the rate of patients aged 80 and over with healthy retinas presenting to outpatient clinics is much lower than for the same age group with AMD. For this reason, the patient population constituting the control group has a relatively lower average age than the study group. As in other studies, we performed an age-matched subgroup analysis in this study to overcome this limitation.^4,5^

Based on the present study and others in the literature, peripheral retinal changes are found in the color and FAF images of AMD patients. It is thought that these peripheral lesions may herald dysfunction at the RPE level. This disease is not exclusively macular but can affect the entire retina at varying rates in all zones, and shows individual differences. In a study evaluating dark adaptation in AMD patients, it was shown that higher patient age, AMD stage, and presence of pseudodrusen were associated with prolonged dark adaptation time.^15^

Only FAF images were evaluated in some studies, while others evaluated both color and FAF images together.^4,5,10,11^ The disadvantage of not evaluating both images together is that in patients with peripheral drusen on color images, FAF images may show hypoautofluorescence, hyperautofluorescence, or no change in autofluorescence. Therefore, the rate of detection of peripheral retinal changes may differ when only FAF images are evaluated.

## Conclusion

In conclusion, we observed a statistically significant difference between AMD patients and the control group in terms of peripheral retinal change detection rates. Our findings are consistent with those of other studies.^4,5,11,12^ The evidence indicates that AMD is not just a macular disease, but rather a disease that can affect the entire retina. Future prospective studies will allow us to determine the relationship between these peripheral retinal changes and patients’ genetic characteristics and their importance in prognosis, diagnosis, and treatment of the disease.

## Figures and Tables

**Table 1:**
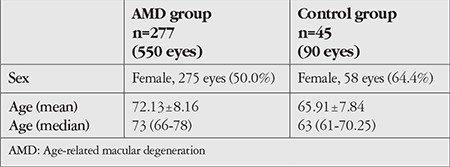
Age and sex distributions of the AMD and control groups

**Table 2:**
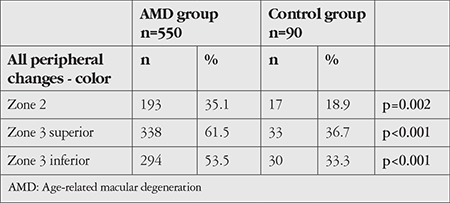
Rates of peripheral retinal changes detected in the color images of patients in the AMD and control groups

**Table 3:**
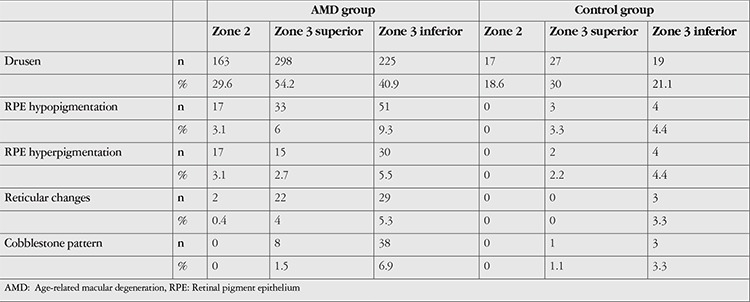
Distribution of all peripheral retinal changes detected in the color fundus images of patients in the AMD and control groups

**Table 4:**
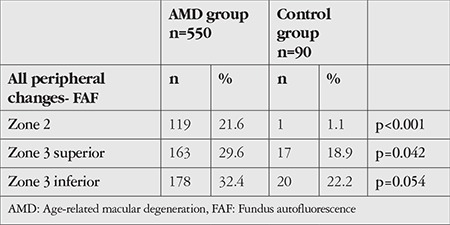
Rates of peripheral autofluorescence changes detected in the fundus autofluorescence images of patients in the AMD and control groups

**Table 5:**
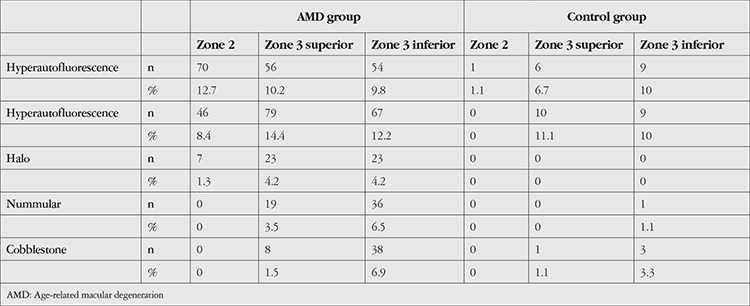
Distribution of all peripheral retinal changes detected in the fundus autofluorescence images of patients in the AMD and control groups

**Table 6:**
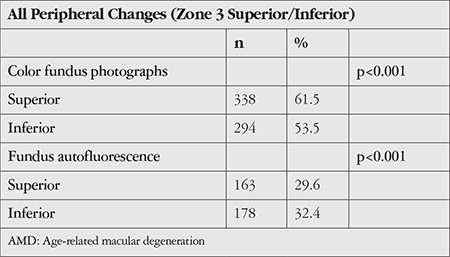
Rates of peripheral retinal and autofluorescence changes in the superior and inferior zone 3 images of patients in the AMD group
